# Predictive performance of dynamic arterial elastance for arterial pressure response to fluid expansion in mechanically ventilated hypotensive adults: a systematic review and meta-analysis of observational studies

**DOI:** 10.1186/s13613-021-00909-2

**Published:** 2021-07-31

**Authors:** Xiaoyang Zhou, Weihao Pan, Bixin Chen, Zhaojun Xu, Jianneng Pan

**Affiliations:** 1 Department of Intensive Care Medicine, HwaMei Hospital, University of Chinese Academy of Sciences, Ningbo, 315000 Zhejiang China; 2 Ningbo Institute of Life and Health Industry, University of Chinese Academy of Sciences, Ningbo, 315000 Zhejiang China; 3 Department of Emergency, Ningbo Yinzhou No.2 Hospital, Ningbo, 315000 Zhejiang China

**Keywords:** Dynamic arterial elastance, Arterial load, Fluid expansion, Hypotension, Diagnostic test accuracy

## Abstract

**Background:**

Dynamic arterial elastance (Ea_dyn_) has been extensively considered as a functional parameter of arterial load. However, conflicting evidence has been obtained on the ability of Ea_dyn_ to predict mean arterial pressure (MAP) changes after fluid expansion. This meta-analysis sought to assess the predictive performance of Ea_dyn_ for the MAP response to fluid expansion in mechanically ventilated hypotensive patients.

**Methods:**

We systematically searched electronic databases through November 28, 2020, to retrieve studies that evaluated the association between Ea_dyn_ and fluid expansion-induced MAP increases in mechanically ventilated hypotensive adults. Given the diverse threshold value of Ea_dyn_ among the studies, we only reported the area under the hierarchical summary receiver operating characteristic curve (AUHSROC) as the primary measure of diagnostic accuracy.

**Results:**

Eight observational studies that included 323 patients with 361 fluid expansions met the eligibility criteria. The results showed that Ea_dyn_ was a good predictor of MAP increases in response to fluid expansion, with an AUHSROC of 0.92 [95% confidence interval (CI) 0.89 to 0.94]. Six studies reported the cut-off value of Ea_dyn_, which ranged from 0.65 to 0.89. The cut-off value of Ea_dyn_ was nearly conically symmetrical, most data were centred between 0.7 and 0.8, and the mean and median values were 0.77 and 0.75, respectively. The subgroup analyses indicated that the AUHSROC was slightly higher in the intensive care unit (ICU) patients (0.96; 95% CI 0.94 to 0.98) but lower in the surgical patients in the operating room (0.72; 95% CI 0.67 to 0.75). The results indicated that the fluid type and measurement technique might not affect the diagnostic accuracy of Ea_dyn_. Moreover, the AUHSROC for the sensitivity analysis of prospective studies was comparable to that in the primary analysis.

**Conclusions:**

Ea_dyn_ exhibits good performance for predicting MAP increases in response to fluid expansion in mechanically ventilated hypotensive adults, especially in the ICU setting.

**Supplementary Information:**

The online version contains supplementary material available at 10.1186/s13613-021-00909-2.

## Background

Fluid expansion is the first-line therapy for the treatment of systemic hypotension which is a very common clinical emergency encountered in the intensive care unit (ICU) [1]. However, fluid expansion does not necessarily increase arterial pressure because fluid-induced changes in arterial pressure depend on both fluid responsiveness and arterial load [2]. Fluid responsiveness is an indicator of great concern for physicians during fluid resuscitation. For those patients who remain hypotensive and fluid-responsive after receiving an arbitrary amount of fluid, clinicians will generally continue to infuse fluid to reach the minimum mean arterial pressure (MAP). However, in the case of depressed vascular tone, MAP may not increase after further fluid therapy despite the increase in cardiac output (CO) [3]. Within this context, aggressive fluid therapy, if aimed at a MAP target, will result in an increased risk of fluid overload. Thus, merely assessing fluid responsiveness to predict MAP increases in response to fluid expansion is not risk free, and arterial load is the other key factor that determines MAP changes in response to fluid administration. Arterial load represents all the extracardiac forces that oppose ventricular ejection, and it comprises different arterial properties (including arterial compliance, total peripheral resistance, etc.), blood viscosity, and arterial wave reflections [4]. Therefore, assessing arterial load before administering more fluids is also essential for hypotensive patients who have received initial fluid resuscitation.

Over the past decade, dynamic arterial elastance (Ea_dyn_) has been extensively considered as a functional parameter of arterial load [3–5]. Since Ea_dyn_ is defined as the ratio of pulse pressure variation (PPV) to stroke volume variation (SVV), it represents the change in arterial pulse pressure for a given change in stroke volume (SV) during a respiratory cycle. Accordingly, Ea_dyn_ describes the dynamic interaction between changes in pressure and flow and dynamically evaluates the changes in arterial load [6–8]. Theoretically, the MAP is more likely to increase when fluid expansion-induced increases in SV cause a proportional or greater increases in arterial pulse pressure during a ventilation cycle (i.e. high Ea_dyn_ value) [3, 6–8]. These rationales underlie the predictive ability of Ea_dyn_ for the MAP response to fluid expansion, which has been demonstrated in many studies [9–12]. However, two recent studies found that Ea_dyn_ failed to predict an increase in MAP after fluid expansion [13, 14]. Considering that conflicting evidence on the predictive value of Ea_dyn_ has not yet been systematically evaluated, we conducted this systematic meta-analysis to assess the predictive performance of Ea_dyn_ for the MAP response to fluid expansion in mechanically ventilated hypotensive patients and investigate the potential influencing factors.

## Methods

This study was reported following the Preferred Reporting Items for a Systematic Review and Meta-analysis of Diagnostic Test Accuracy [15]. We registered the review protocol with the International Prospective Register of Systematic Reviews (PROSPERO; CRD42020223455) prior to study selection. Given the nature of this review article, an institutional review board and written informed consent were not required.

### Data sources and search strategy

Two reviewers (Zhou X and Pan W) independently and systematically searched on the PubMed, Embase, Web of Science, and Cochrane Central Register of Controlled Trials from database inception to November 28, 2020, to retrieve studies that evaluated the association between Ea_dyn_ and MAP increases associated with fluid expansion in mechanically ventilated hypotensive adults, without any date or language restrictions. The bibliographies of relevant publications were also searched manually to further identify relevant articles. The detailed search strategies are listed in Additional file 1: Table S1.

### Inclusion and exclusion criteria

We established stringent eligibility criteria for screening relevant studies. Studies that met all of the following criteria were eligible: (1) observational studies on mechanically ventilated adults (age > 18 years) who were hypotensive (MAP < 65 mmHg) or receiving norepinephrine (NE) to maintain arterial pressure; (2) fluid expansion administration was planned by the clinicians in charge; (3) MAP changes before and after fluid expansion were assessed and considered the reference gold standard to define MAP responsiveness (regardless of the threshold value) and Ea_dyn_ was measured as the index test; and (4) sufficient information was reported to construct a 2 × 2 contingency table. Studies that met one of the following criteria were ineligible: (1) studies that enrolled patients with spontaneous breathing efforts or normotensive patients; (2) studies that did not report the diagnostic performance of Ea_dyn_; and (3) conference abstracts without full text.

### Study selection and data extraction

All searched records were independently screened by two authors (Chen B and Xu Z), who reviewed the titles and abstracts after deduplication. The same two authors independently reviewed the full text of the selected records for eligibility. Disagreements were resolved by discussion, and a third reviewer (Pan J) was involved if necessary. The reasons for excluding the ineligible studies are presented in Additional file 1: Table S2.

Two independent authors (Chen B and Xu Z) extracted data from each study using a customized extraction form. The extracted data included the study characteristics, patient characteristics, and diagnostic accuracy measures of the index test. We calculated the true positive, false positive, false negative, and true negative values to construct the 2 × 2 contingency table according to the sensitivity, specificity, and sample size in each study. In studies that did not report sensitivity or specificity information, we returned to the original receiver operating characteristic (ROC) curve to identify the optimal cut-off point and estimate its corresponding sensitivity and specificity. In addition, we contacted the authors of the included studies to obtain the missing data of interest. Any disagreements were resolved by a joint review of the full text to reach a consensus.

### Quality assessment

The risk of bias of each included study was evaluated independently by two authors (Zhou X. and Pan J.) using the Quality Assessment of Diagnostic Accuracy Studies (QUADAS)-2 tool [16]. The QUADAS-2 consists of 4 domains: patient selection, index test, reference standard, and flow and timing, and the first three domains were also assessed for applicability concerns. Disagreements were resolved by consensus.

### Statistical analysis

Initially, the derived estimates of sensitivity and specificity from each study were plotted on forest plots and ROC space to explore between‐study variations. Due to the expected between‐study variations, both the bivariate model [17] and hierarchical summary ROC (HSROC) model [18] were adopted to calculate summary estimates of diagnostic accuracy measures and fit an HSROC curve [19]. The bivariate model includes a correlation parameter that allows for the expected trade‐off in sensitivity and specificity because the test positivity threshold varied across studies [19]. The HSROC model incorporates both sensitivity and specificity while taking into account the possible correlation between them. Between-study heterogeneity was evaluated using Cochran’s *Q* test and *I*^2^ statistics. The threshold effect was evaluated through a visual inspection of the HSROC curve and calculation of the Spearman correlation coefficient (*ρ*) between the sensitivity and false-positive rate. As the cut-off value of Ea_dyn_ varied across the included studies, we only presented the area under the HSROC curve (AUHSROC) as the main measure of diagnostic accuracy. We avoided using the summary sensitivity and specificity as the main accuracy measure because estimates for certain notional unspecified average of different thresholds are clinically uninterpretable [19]. All data syntheses were performed using the MIDAS and METANDI modules in Stata/SE 15.0 software (Stata-Corp, College Station, TX, USA). A two-tailed *P* < 0.05 was considered statistically significant.

We constructed a scatter plot to observe the distribution, dispersion, and central tendency of the cut-off value of Ea_dyn_ in all studies that reported such data. We also calculated the mean and median cut-off values of Ea_dyn_ to estimate the optimal threshold value for predicting the MAP response to fluid expansion. We conducted subgroup analyses according to the classification of patients (ICU patients or surgical patients in the operating room), fluid type (colloid or crystalloid), and measurement technique for SVV (arterial waveform analysis or oesophageal Doppler) because these factors might have impacts on the predictive performance of Ea_dyn_. To confirm the stability of the present study, we conducted two sensitivity analyses by restricting the analyses to prospective studies and excluding the outliers identified by drawing a Galbraith plot. A Bayesian nomogram was constructed to calculate the posttest probability to facilitate the interpretation of the clinical utility of Ea_dyn_ for predicting the MAP response to interventions. Publication bias was assessed by Deeks’ funnel plot asymmetry test [20].

## Results

### Study selection

**Fig. 1:**
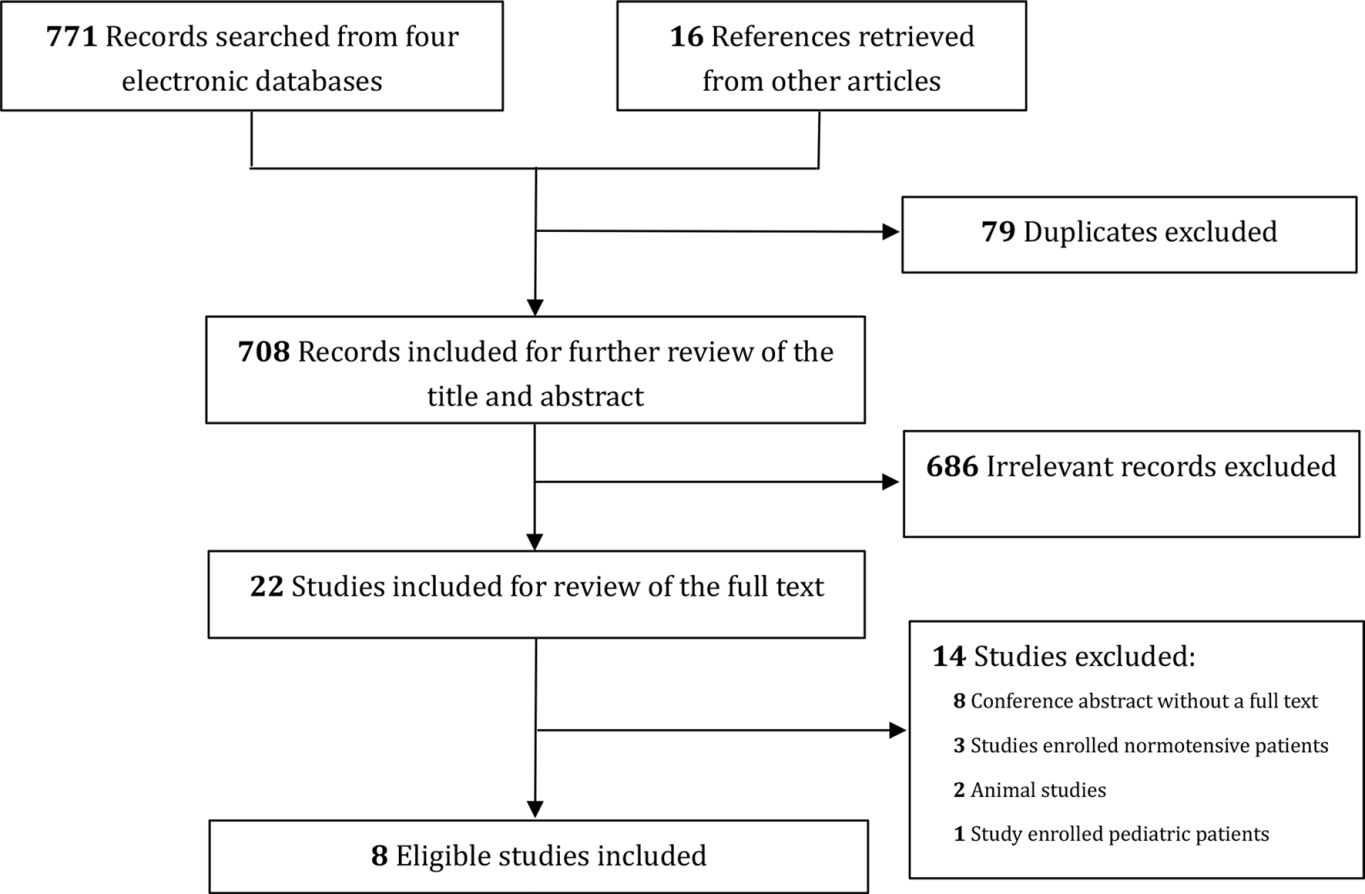
Flowchart of study selection

The database search yielded a total of 771 records. Additional 16 records were retrieved from other publications. After excluding 79 duplicates and 686 irrelevant records, 22 records were reviewed for the full text. Finally, eight studies [9–14, 21, 22] that met the eligibility criteria were included for the quantitative analysis. Figure 1 depicts the study selection process in detail.

### Characteristics of included studies

Details pertaining to the study and patient characteristics are described in Table 1. All the included studies [9–14, 21, 22] were published after 2011 and included a total of 323 patients. Among the included studies, six [9–12, 14, 22] were prospectively designed, and two [13, 21] were retrospectively designed. In addition, five studies [9, 10, 14, 21, 22] were conducted in the ICU and mainly recruited medical patients, and the remaining three studies [11–13] were performed in the operating room and recruited surgical patients. Interestingly, none of the latter three studies [11–13] that conducted in the operating theatre used vasopressor during fluid expansion. The fluid volume and infusion duration during the fluid challenge test largely varied among the included studies. Colloid fluid was administered in four studies [9, 11, 13, 21], and crystalloid fluid was infused in the other four studies [10, 12, 14, 22]. All patients in six studies [9–12, 14, 21] had a preload dependency. However, only some of the patients in the remaining two studies [13, 22] had a preload dependency. The threshold for defining MAP responsiveness was 15% in 4 studies [9, 11, 13, 21] and 10% in three studies [10, 12, 14]. One study [22] defined MAP responders as showing a restoration of the MAP to > 65 mmHg after fluid expansion. All the included studies measured the PPV using arterial waveform analysis, and five studies [9, 11, 13, 14, 22] used arterial waveform analysis to estimate the SVV. However, the SVV was measured by oesophageal Doppler in two studies [10, 12], and by pulse indicator continuous cardiac output (PICCO) in one study [21]. The diagnostic accuracies of each study are presented in Table 2.

**Table 1:** Characteristics of the included studies

Study no.	Author/year	Design, setting, location, and publication language	Subjects	Preload dependency	Vasopressor used (*n*)	Sample size	No. of fluid expansions	Age (mean, years)	Male (n, %)	MAP at baseline (mean, mm Hg)	Tide volume (mean or median mL/kg)	PEEP (mean or median, cm H_2_O)
1	Monge García/2011	Prospective study; mixed ICU; Spain; English	Mechanically ventilated hypotensive patients with acute circulatory failure	Yes	Yes (12)	25	25	61	15 (60.0)	58	8.6	7.9
2	Gong/2013	Retrospective study; ICU; China; Chinese	Mechanically ventilated patients with distributive or hypovolemic shock	Yes	Yes (unknown)	32	32	66	NR	NR	> 8	NR
3	Monge García/2014	Prospective study; mixed ICU; Spain; English	Mechanically ventilated patients with acute circulatory failure	Yes	Yes (30)	53	80	63	31 (58.5)	71	8	8
4	Seo/2015	Prospective study; operating room; Republic of Korea; English	Mechanically ventilated hypotensive patients receiving robot-assisted laparoscopic prostatectomy	Yes	No	39	39	64	39 (100)	64	8	8
5	Lanchon/2017	Retrospective study; operating room; France; English	Mechanically ventilated hypotensive surgical patients	Yes (part of patients)	No	51	51	62	31 (60.8)	56	8.2	3
6	de Courson/2019	Prospective study; 2 operating rooms; France; English	Mechanically ventilated hypotensive surgical patients	Yes	No	56	56	57	21 (37.5)	59	7.4	5
7	Guarracino/2019	Prospective study; ICU; USA; English	Mechanically ventilated hypotensive patients with sepsis or septic shock	Yes (part of patients)	Yes (unknown)	55	55	69	34 (61.8)	57	6–8	NR
8	Luetrakool/2020	Prospective study; medical ICU; Thailand; English	Mechanically ventilated ARDS patients with acute circulatory failure	Yes	Yes (12)	12	23	61	NR	61	7.2	11.5

Study no.	Author/year	Fluid type, volume, and duration	No. of MAP responder (*n*, %)	Threshold for MAP responsiveness	Technique to measure SVV	Fluid responsiveness
1	Monge García/2011	Synthetic colloid (6% hydroxyethyl starch), 500 mL, over 30 min	16 (64)	MAP increase ≥ 15%	Arterial waveform analysis	Presence of a stable value of SVV ≥ 10%
2	Gong/2013	Colloid, 500 mL, within 30 min	19 (59.4)	MAP increase ≥ 15%	PICCO	SVV > 10%
3	Monge García/2014	Normal saline, 500 mL, within 30 min	33 (41.2)	MAP increase ≥ 10%	Oesophageal Doppler	Cardiac output increase ≥ 10% after a 2-min leg-raising manoeuvre
4	Seo/2015	6% Hydroxyethyl starch, 500 mL, over 20 min	17 (43.6)	MAP increase ≥ 15%	Arterial waveform analysis	Maintenance of SVV > 10% for > 10 min
5	Lanchon/2017	6% starch, 500 mL, over 10 min	17 (33.3)	MAP increase ≥ 15%	Arterial waveform analysis	Increase in stroke volume ≥ 15% after volume expansion
6	de Courson/2019	0.9% Saline, 250 mL, over 10 min	21 (37.5)	MAP increase ≥ 10%	Oesophageal Doppler	SVV > 10%, assessed by using oesophageal Doppler
7	Guarracino/2019	0.9% Saline, 30 mL/kg, within 3 h of enrolment	35 (63.6)	MAP restored to > 65 mmHg	Arterial waveform analysis	Cardiac index increased by > 15% after fluid expansion
8	Luetrakool/2020	Crystalloid, 500 mL, over 15 min	9 (39.1)	MAP increase ≥ 10%	Arterial waveform analysis	Passive leg-raising test: cardiac output (CO) increase of 10% or more, or Mini-fluid challenge test: increase in velocity time integral by 10% or more after 100 mL crystalloid infusion over one minute

*No.* number, *MAP* mean arterial pressure, *NE* norepinephrine, *PEEP* positive end-expiratory pressure, *ICU* intensive care unit, *SVV* stroke volume variation, *NR* no record, *PICCO* pulse indicator continuous cardiac output

**Table 2:** Detailed diagnostic accuracy of dynamic arterial elastance in each included study

Study no.	Author/year	AUROC	Sensitivity (%)	Specificity (%)	Cut-off value	True positive	False positive	False negative	True negative
1	Monge García/2011	0.986	93.75	100	0.89	15	0	1	9
2	Gong/2013	0.95	89.5	92.3	0.85	17	1	2	12
3	Monge García/2014	0.94	90.9	91.5	0.73	30	4	3	43
4	Seo/2015	0.81	70.6	86.4	0.74	12	3	5	19
5	Lanchon/2017*	0.54	58.8	52.9	NR	10	16	7	18
6	de Courson/2019	0.71	76.2	60.0	0.65	16	14	5	21
7	Guarracino/2019	0.954	100	95	0.76	35	1	0	19
8	Luetrakool/2020*	0.67	55.6	78.6	NR	5	3	4	11

*No.* number, *MAP* mean arterial pressure, *AUROC* area under the receive operator characteristic curve, *NR* no record

* The study failed to identify the predictive ability of dynamic arterial elastance. These two studies only reported the AUROC value. Thus, we returned to the original ROC curve in their article to identify the cut-off point and estimate its corresponding sensitivity and specificity

### Methodological quality of included studies

The methodological quality of each study is summarized in Table 3. None of the included studies was of high methodological quality. In the patient selection domain, four studies [12, 13, 21, 22] were at high risk of bias because they did not include a consecutive or random series of participants, and one study [10] had a high applicability concern because some patients were receiving NE to maintain a MAP > 65 mmHg before fluid expansion. Because the reference standard in one study [22] likely led to an incorrect classification of the target condition, they were judged as having a high risk of bias and high applicability concern in the reference standard domain.

**Table 3:**
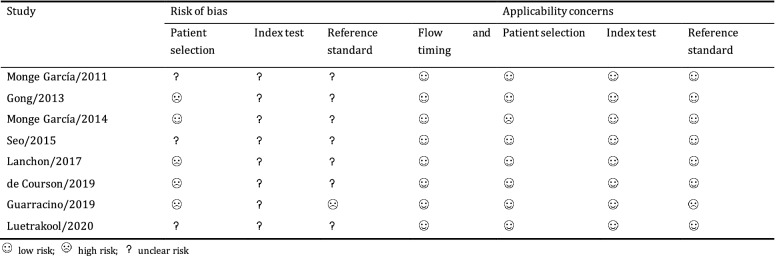
Methodological quality of each included study

### Prediction of the fluid expansion-induced increases in MAP

**Fig. 2:**
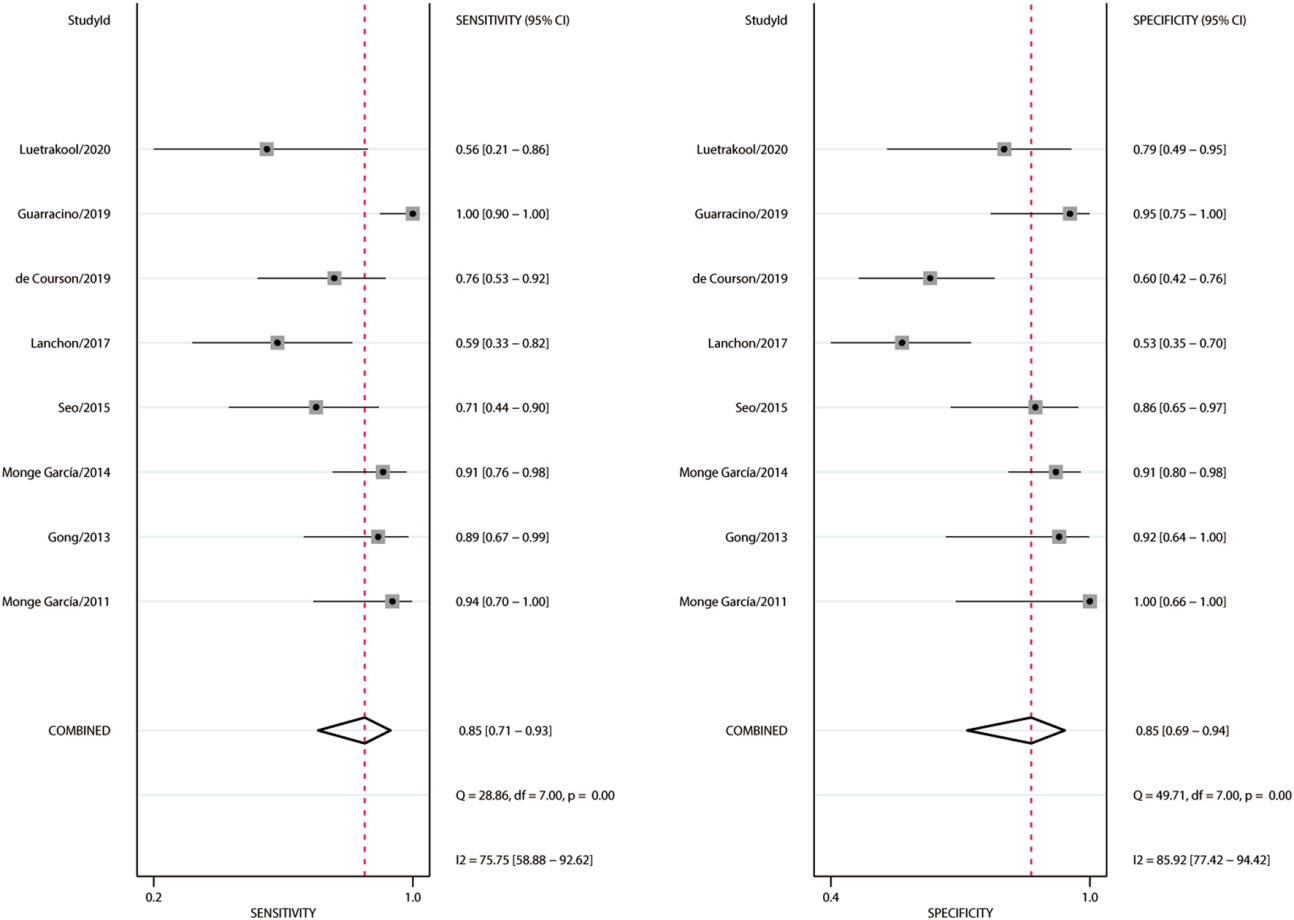
Forest plot of sensitivity and specificity of dynamic arterial elastance for predicting the MAP response to fluid expansion

**Fig. 3:**
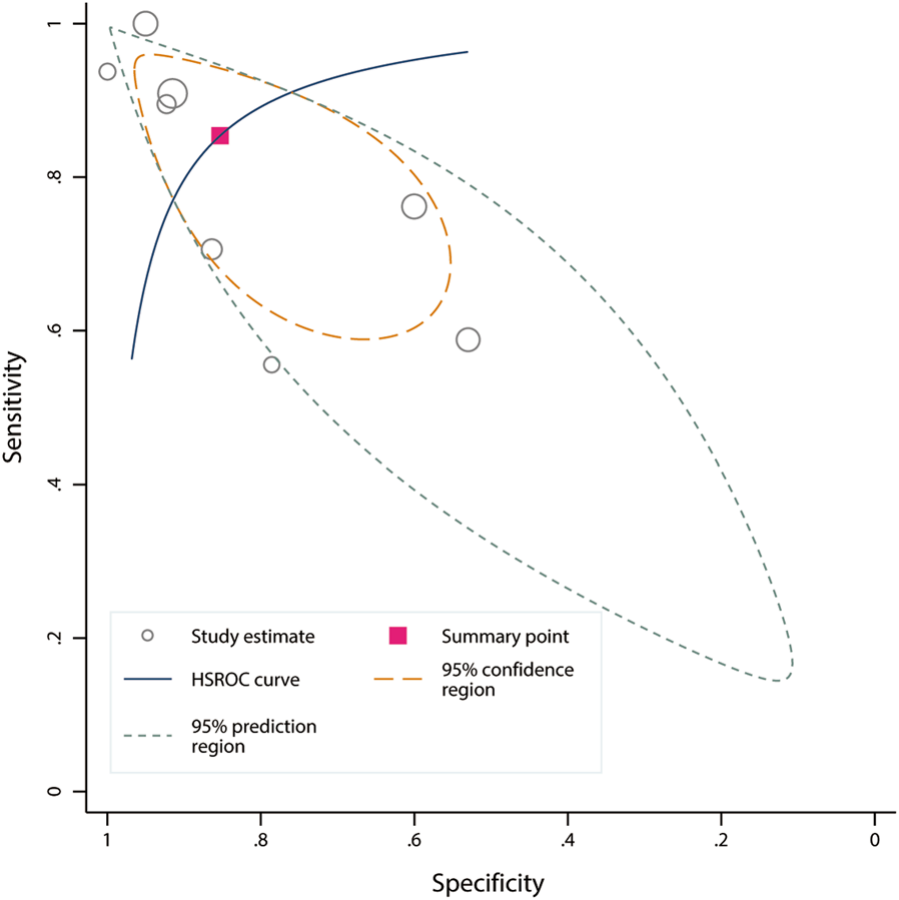
HSROC curve of dynamic arterial elastance for predicting the MAP response to fluid expansion. The size of the circles indicates the weight of the individual studies. The area under the hierarchical summary receiver operating curve was 0.92 (95% CI 0.89 to 0.94)

**Fig. 4:**
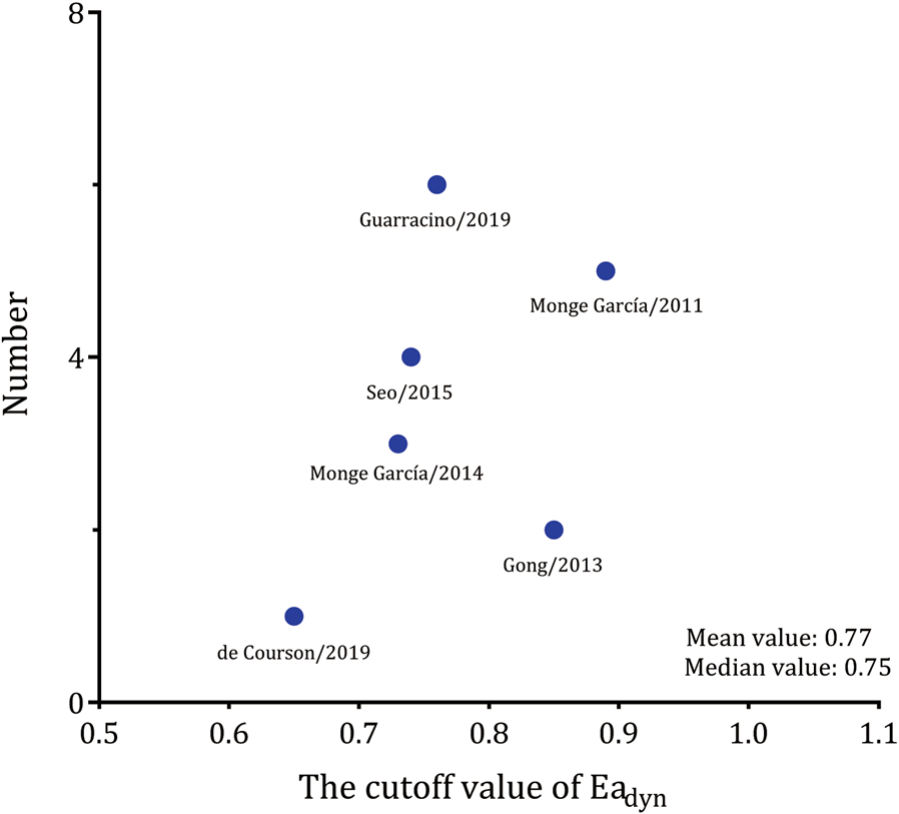
Scatter plot of the cut-off value of dynamic arterial elastance. The distribution was nearly conically symmetrical, most data were centred between 0.7 and 0.8, and the mean and median values were 0.77 and 0.75, respectively

A total of 361 fluid expansions were administered, and 162 (46.3%) of them were MAP responders. Estimates of sensitivity ranged from 55.6 to 100%, and estimates of specificity ranged from 60.0 to 100% (Fig. 2). Heterogeneity between studies was assessed with a Cochran *Q* statistic of 0.061 (*P* = 0.485) and an overall *I*^2^ of 0%. However, significant heterogeneities were found for the pooled sensitivity and specificity (Fig. 2). The threshold effect across the included studies was confirmed by visual inspection of the HSROC curve (Fig. 3) and the Spearman correlation coefficient (*ρ* = 1.0), indicating that all heterogeneities were caused by the threshold effect. The AUHSROC was 0.92, with corresponding 95% confidence interval (CI) of 0.89 to 0.94. Six studies [9–12, 21, 22] reported the cut-off value of Ea_dyn_, which ranged from 0.65 to 0.89 (Table 2). According to the scatter plot (Fig. 4), we observed that the distribution of the cut-off value of Ea_dyn_ was nearly conically symmetrical and most data were centred between 0.7 and 0.8, which might represent the ‘gray zone’ for the prediction of MAP increases after fluid expansion. The mean cut-off value was 0.77 with a standard deviation of 0.09, and the median value was 0.75.

### Subgroup analysis and sensitivity analysis

The subgroup analysis suggested that the AUHSROC was slightly higher in ICU patients (0.96, 95% CI 0.94 to 0.98) than in surgical patients in the operating room (0.72, 95% CI 0.67 to 0.75). However, the AUHSROC did not differ between subgroups of colloid fluid and crystalloid fluid. The measurement technique also did not affect the diagnostic accuracy of Ea_dyn_ (Table 4).

**Table 4:** Effect estimates of dynamic arterial elastance for predicting the mean arterial pressure response to fluid expansion

Variables	Subgroups	No. of studies	AUHSROC	Sensitivity (%)	Specificity (%)	Diagnostic odds ratio
Primary analysis	All studies	8	0.92 (0.89, 0.94)	0.85 (0.71, 0.93)	0.85 (0.69, 0.94)	22.8 (5.5, 93.6)
Classification of patients	ICU patients	5	0.96 (0.94, 0.98)	0.92 (0.75, 0.98)	0.92 (0.82, 0.97)	76.3 (13.0, 448.9)
	Surgical patients in the operating room	3	0.72 (0.67, 0.75)	0.69 (0.56, 0.80)	0.66 (0.48, 0.81)	4.5 (1.3, 14.9)
Fluid type	Colloid	4	0.90 (0.87, 0.93)	0.81 (0.60, 0.92)	0.88 (0.58, 0.98)	19.4 (2.2, 174.1)
	Crystalloid	4	0.93 (0.90, 0.95)	0.89 (0.65, 0.97)	0.85 (0.62, 0.95)	30.0 (3.4, 248.7)
Measurement technique	Arterial waveform analysis	5	0.92 (0.90, 0.94)	0.85 (0.58, 0.96)	0.87 (0.61, 0.97)	19.1 (2.6, 139.8)
	Oesophageal Doppler	2	0.90 (0.87, 0.92)	0.85 (0.66, 0.94)	0.80 (0.49, 0.94)	21.8 (1.0, 458.4)
	PICCO	1	–	–	–	–
Sensitivity analyses	Excluding the outlier*	7	0.88 (0.85, 0.91)	0.80 (0.68, 0.89)	0.83 (0.66, 0.92)	15.3 (4.0, 59.2)
	Excluding 2 retrospective studies	6	0.94 (0.91, 0.96)	0.88 (0.71, 0.95)	0.87 (0.71, 0.95)	30.1 (6.5, 138.6)

*MAP* mean arterial pressure, *ICU* intensive care unit, *AUHSROC* area under hierarchical summary receiver operating characteristic curve, *PICCO* pulse indicator continuous cardiac output

* The study by Guarracino et al. was identified as the outlier based on a Galbraith plot

The study by Guarracino et al. [22] was identified as the outlier based on a Galbraith plot (Additional file 1: Figure S1). After excluding the outlier, the sensitivity analysis still suggested a good predictive accuracy of Ea_dyn_, although the AUHSROC was slightly lower (Table 4). When restricting analyses to prospective studies, the AUHSROC was comparable to the primary analysis (Table 4). Thus, the two sensitivity analyses confirmed the robustness of our results. As shown in the Bayes nomogram (Additional file 1: Figure S2), if an average-risk population had an assumed pretest probability of 50%, Ea_dyn_ increased the probability of MAP responders to 85% when the test result was positive and decreased the probability to 15% when the test result was negative. We found no significant publication bias by Deeks’ funnel plot asymmetry test (*P* = 0.66) (Additional file 1: Figure S3).

## Discussion

This systematic meta-analysis suggested a good diagnostic accuracy of Ea_dyn_ for predicting MAP increases in response to fluid expansion in mechanically ventilated hypotensive adults. The diagnostic accuracy of Ea_dyn_ was slightly improved in the ICU patients but decreased in surgical patients in the operating room. The fluid type and technique for measuring SVV seemed to have no impact on the diagnostic accuracy of Ea_dyn_.

Based on the subgroup analyses, one could expect that the diagnostic accuracy of Ea_dyn_ may be reduced in surgical patients in the operating theatre, even though the pooled results from limited patients did not lead to a firm conclusion. Interestingly, we also found that most patients in studies conducted in the ICU [9, 10, 14, 21, 22] received vasopressors; however, none of the studies conducted in the operating theatre [11–13] used vasopressors during fluid expansion. This finding is not surprising because patients admitted to the ICU setting commonly suffer from various cardiovascular disorders, such as sepsis-induced cardiomyopathy and vasoplegia [23, 24], and surgical patients are generally healthier than ICU patients from the perspective of cardiovascular function. Thus, we supposed that vasopressors used during fluid expansion might be the main contributor to the different predictive performances of Ea_dyn_ between ICU patients and surgical patients. Pulse pressure is a pulsatile haemodynamic index that results from the interaction of left ventricular mechanical work with the arterial tree [25]. Thus, Ea_dyn_ (i.e. the PPV/SVV ratio) likely depends on the pulsatile components of arterial load, primarily on arterial compliance [26, 27], due to the oscillatory nature of the arterial pressure-flow relationship. Previous studies demonstrated that vasopressors can restore arterial compliance in hypotensive patients with vasoplegia [7, 27]. Moreover, there is a growing body of evidence suggesting that arterial compliance is fixed and not altered by fluid expansion in patients receiving norepinephrine [10, 28]. However, in hypotensive patients free of vasopressors, fluid expansion could change arterial compliance during resuscitation [12]. Hence, Ea_dyn_ before fluid administration could reflect the baseline arterial tone and track the fluid-induced changes in arterial pressure in patients receiving vasopressors, whereas fluid expansion might change arterial compliance in patients who did not receive vasopressors and thus decrease the ability of Ea_dyn_ to reflect the baseline arterial tone. Additionally, pneumoperitoneum in abdominal surgery was another factor influencing the diagnostic accuracy of Ea_dyn_ [11, 12] because the increased abdominal pressure induced by pneumoperitoneum might cause significant circulatory perturbations [29], and increased plasma noradrenaline and changes in total peripheral resistance have been associated with pneumoperitoneum [30]. Accordingly, clinicians should be cautious in applying Ea_dyn_ to predict the arterial pressure response to fluid in surgical patients in the operating room who are free of vasopressors.

Our findings suggested that the cut-off value of Ea_dyn_ largely differed between the included studies. The diverse threshold values potentially resulted from the varied arterial pulse-contour algorithm used and the distinct diseases studied. The SVV obtained from different devices on the same patient may be dissimilar because the results for this parameter depend on the algorithm used [31]. The underlying pathophysiological mechanisms are largely different between medical and surgical diseases and can affect arterial tone, which likely leads to diverse cut-off values. To facilitate a better understanding of the clinical significance of Ea_dyn_ in the decision-making process, we observed the distribution, dispersion, and central tendency of the cut-off values to identify the ‘gray zone’, which is anticipated to avoid the binary constraint of a “black-or-white” decision of the ROC curve and fit the reality of clinical or screening practice [32]. The results indicated that the distribution of the cut-off values was nearly conically symmetrical, and most data were centred between 0.7 and 0.8, which might represent the ‘uncertain zone’ for the prediction of fluid-induced MAP changes. Thus, if the measured baseline Ea_dyn_ is above 0.8, fluid expansion would increase arterial pressure in hypotensive patients with preload dependency. Conversely, if the measured baseline Ea_dyn_ is below 0.7, fluid administration might not increase arterial pressure despite the increase in CO. In this case, NE should be used early to maintain arterial pressure. The combinational use of fluid expansion and NE would likely decrease the amount of fluid administered and reduce the risk of fluid overload. Overall, Ea_dyn_ may be a reliable haemodynamic indicator that can help physicians choose the optimal therapeutic strategy without requiring complicated monitoring devices.

This meta-analysis has several strengths. To our knowledge, this is the first meta-analysis to systematically assess the predictive performance of Ea_dyn_ for the MAP changes associated with volume expansion. We used the AUHSROC instead of pooled sensitivity and specificity as the main measure of diagnostic accuracy because the latter measures might lead to misleading interpretations of our results when different cut-off values occur between the included studies. To reduce the heterogeneity among the included studies, we established stringent eligibility criteria and only mechanically ventilated hypotensive adults were included in this study. Additionally, we conducted several meaningful subgroup analyses to explore potential influencing factors. However, several limitations of our study should be recognized when interpreting the findings. First, the sample size and study numbers in our study were limited. As a result, the limited statistical power hampered us from drawing a firm conclusion. Moreover, studies with small sample sizes may overestimate the effect sizes [33]. Thus, the current findings need to be further confirmed in larger studies. Second, all of the included studies were at high risk of bias, and some studies also had high concerns regarding applicability. Consequently, none of the included studies was judged as having high methodological quality. These methodological shortcomings might intrinsically lead to a potential bias in our results and thereby restrict the validity and applicability of our findings. Accordingly, the findings in this study should be interpreted with caution. Last, the heterogeneities among the included studies might partly be attributed to the different techniques used to measure SVV. As the reliability of Ea_dyn_ primarily depends on the robustness of the SV estimation methods [31], the varied pulse-contour analysis methods used (with different algorithms for estimating SVV) might contribute to the diverse threshold values of Ea_dyn_, which were the main sources of observed heterogeneities in this meta-analysis. Nevertheless, the subgroup analysis suggested that the predictive performance of Ea_dyn_ was not influenced by the measurement techniques. Consequently, we have reasons to believe that the calculated Ea_dyn_ is valid as long as the algorithm used to estimate SV is trustworthy.

## Conclusion

In mechanically ventilated hypotensive adults, the measurement of Ea_dyn_ is a useful approach for predicting MAP changes in response to fluid expansion, especially in the ICU setting. The fluid type and technique for measuring SVV may not be associated with the predictive performance of Ea_dyn_. Because of the small sample size and the low methodological quality of the included studies, larger studies with high methodological quality are warranted in the future to validate the applicability of Ea_dyn_ in clinical practice.

## Supplementary Information

**Additional file 1: Table S1.** Detailed search strategy for each database. **Table S2.** Ineligible studies and the reasons for exclusion. **Figure S1.** Galbraith plot to identify the outlier. **Figure S2.** Bayes nomogram of dynamic arterial elastance for the prediction of mean arterial response to fluid expansion. **Figure S3.** Deeks’ funnel plot to assess the publication bias.

## Data Availability

All data generated or analysed during this study are included in this published article (and its additional information files).

## Abbreviations

ICU: Intensive care unit
Ea_dyn_: Dynamic arterial elastance
MAP: Mean arterial pressure
NE: Norepinephrine
CO: Cardiac output
SV: Stroke volume
PPV: Pulse pressure variation
SVV: Stroke volume variation
PICCO: Pulse indicator continuous cardiac output
ROC: Receiver operating characteristic
HSROC: Hierarchical summary ROC
AUHSROC: The area under the HSROC curve
CI: Confidence interval
