# Supplementary material for: Predictive performance of dynamic arterial elastance for arterial pressure response to fluid expansion in mechanically ventilated hypotensive adults: a systematic review and meta-analysis of observational studies
Source: Ann Intensive Care. 2021 Jul 31;11:119. doi: 10.1186/s13613-021-00909-2 (PMC8325731; doi:10.1186/s13613-021-00909-2)
Supplement: Supplementary file 1 — Additional file 1: Table S1. Detailed search strategy for each database. Table S2. Ineligible studies and the reasons for exclusion. Figure S1. Galbraith plot to identify the outlier. Figure S2. Bayes nomogram of dynamic arterial elastance for the prediction of mean arterial response to fluid expansion. Figure S3. Deeks’ funnel plot to assess the publication bias. [file 13613_2021_909_MOESM1_ESM.docx]

**Predictive performance of dynamic arterial elastance for arterial pressure response to fluid expansion in mechanically ventilated hypotensive adults – a systematic review and meta-analysis of observational studies**

**ADDITIONAL FILE 1.**

**Table of contents**

[Table S1. Detailed search strategy for each database 2](#_Toc74082366)

[Table S2. Ineligible studies and the reasons for exclusion 3](#_Toc74082367)

[Figure S1. Galbraith plot to identify the outlier 5](#_Toc74082368)

[Figure S2. Bayes nomogram of dynamic arterial elastance for the prediction of mean arterial response to fluid expansion 6](#_Toc74082369)

[Figure S3. Deeks’ funnel plot to assess the publication bias 7](#_Toc74082370)

# Table S1. Detailed search strategy for each database

| **PUBMED** (447 records) | | |
| --- | --- | --- |
| **#** | **Details** | **Records** |
| 1 | "Arterial Pressure"[Mesh] (5780 records) | 5780 |
| 2 | (((((((Arterial Pressure*) OR (Arterial Tension*)) OR (Blood Pressure*)) OR (Arterial Blood Pressure*)) OR (Mean Arterial Pressure*)) OR (Mean Aortic Pressure*)) OR (Radial artery pressure*)) OR (Femoral artery pressure*) | 670504 |
| 3 | #1 OR #2 | 670504 |
| 4 | (((Dynamic arterial elastance) OR (Pulse pressure variation/Stroke volume variation)) OR (PPV/SVV ratio)) OR (PPV/SVV) | 1140 |
| 5 | #3 AND #4 Filters: Humans | 447 |
| **EMBASE** (135 records) | | |
| **#** | **Details** | **Records** |
| 1 | arterial AND pressure* OR (arterial AND tension*) OR (blood AND pressure*) OR (arterial AND blood AND pressure*) OR (mean AND arterial AND pressure*) OR (radial AND artery AND pressure) OR (femoral AND artery AND pressure) | 927721 |
| 2 | dynamic AND arterial AND elastance | 147 |
| 3 | #1 AND #2 | 135 |
| **Web of Science** (169 records) | | |
| **#** | **Details** | **Records** |
| 1 | TS: (Arterial Pressure*) OR TS: (Arterial Tension*) OR TS: (Blood Pressure*) OR TS: (Arterial Blood Pressure*) OR TS: (Mean Arterial Pressure*) | 458645 |
| 2 | TS: (Dynamic arterial elastance) OR TS: (Pulse pressure variation/Stroke volume variation) OR TS: (PPV/SVV ratio) OR TS: (PPV/SVV) | 193 |
| 3 | #1 AND #2 | 169 |
| **Cochrane Central Register of Controlled Trials** (20 records) | | |
| 1 | MeSH descriptor: [Arterial Pressure] explode all trees | 415 |
| 2 | (Arterial Pressure*):ti,ab,kw OR (Arterial Tension*):ti,ab,kw OR (Blood Pressure*):ti,ab,kw OR (Arterial Blood Pressure*):ti,ab,kw OR (Mean Arterial Pressure*):ti,ab,kw | 112178 |
| 3 | #1 OR #2 | 112178 |
| 4 | (Dynamic arterial elastance):ti,ab,kw | 27 |
| 5 | #3 AND #4 | 20 |

# Table S2. Ineligible studies and the reasons for exclusion

| **Reasons for exclusion** | **Studies** |
| --- | --- |
| Conference abstract without a full text | Bastoni/2016 [1]; Di Tomasso/2014 [2]; El-Sayed/2018 [3]; Guinot/2014 [4]; Monge García/2010 [5]; Monge Garcia/2013 [6]; Niyatiwatchanchai/2018 [7]; Theerawit/2016 [8] |
| The enrolled patients had a normal blood pressure before intervention | Lee/2020 [9]; Wu/2016 [10]; Cecconi/2015 [11] |
| Animal studies | Monge Garcia/2020 [12]; Monge García/2017 [13]; |
| Study enrolled pediatric patients | Lee/2020 [14] |

**Reference:**

1. Bastoni D, Aya H, Toscani L, Pigozzi L, Rhodes A, Cecconi M. Dynamic arterial elastance calculated with lidcoplus monitor does not predict changes in arterial pressure after a fluid challenge in postsurgical patients. Critical Care. 2016; 20 SUPPL. 2.
2. Di Tomasso N, Vanoni M, Mellinghoff J, Grounds R.M, Rhodes A, Cecconi M. PPV and SVV ratio and map response to a fluid challenge in SV responsive mechanically ventilated patients. A preliminary study to find a functional haemodynamic parameter for fluid responsiveness prediction. Intensive Care Med. 2014; 40:1 SUPPL. 1 (S178).
3. El-Sayed M, Salah Hammad Ali S, Mohamady El-Demerdash Hassan A, Elsaied Abdelrahman A., Mukhtar A. Dynamic arterial elastance as a predictor of arterial blood pressure response to fluid administration in septic patients: A prospective observational study. Intensive Care Med Experimental. 2018; 6 Supplement 2.
4. Guinot PG, Bernard E, Levrard M, Zogheib E, Dupont H, Lorne E. The measure of a dynamic arterial elastance predicted the decrease in blood pressure when sewage of noradrenaline in septic shock. Annals Francaises D Anesthesie De Reanimation. 2014.33(2): A222.
5. Monge García M.I, Gil Cano A, Gracia Romero M. Dynamic assessment of arterial elastance to predict arterial pressure response to volume loading in preload-dependent patients. Intensive Care Med. 2010; 36 SUPPL. 2 (S362).
6. Monge Garcia M.I, Gracia Romero M, Gil Cano A., Rhodes A, Grounds R.M., Maurizio C. Dynamic arterial elastance is a predictor of arterial pressure changes to volume administration in fluid responder patients. Intensive Care Med. 2013; 39 SUPPL. 2 (S233).
7. Niyatiwatchanchai N, Liwsrisakun C, Chittawatanarat K, Theerakittikul T, Limsukon A, Tajarernmuang P, Pothirat C. Dynamic arterial elastance to predict mean arterial pressure after decreasing norepinephrine dosage in septic shock. Intensive Care Med Experimental. 2018 ;6 Supplement 1.
8. Theerawit P, Morasert T, Sutherasan Y. Diastolic blood pressure, static arterial elastance, dynamic arterial elastance and arterial resistance during fluid challenge in septic shock: A pilot study. Intensive Care Med Experimental. 2016; 4 Supplement 1.
9. Lee CT, Lee TS, Chiu CT, Teng HC, Cheng HL, Wu CY. Mini-fluid challenge test predicts stroke volume and arterial pressure fluid responsiveness during spine surgery in prone position: A STARD-compliant diagnostic accuracy study. Medicine (Baltimore). 2020;99(6):e19031.
10. Wu CY, Cheng YJ, Liu YJ, Wu TT, Chien CT, Chan KC; NTUH Center of Microcirculation Medical Research (NCMMR). Predicting stroke volume and arterial pressure fluid responsiveness in liver cirrhosis patients using dynamic preload variables: A prospective study of diagnostic accuracy. Eur J Anaesthesiol. 2016;33(9):645-52.
11. Cecconi M, García MIM, Romero MG, Mellinghoff J, Caliandro F, Grounds RM, et al. The use of pulse pressure variation and stroke volume variation in spontaneously breathing patients to assess dynamic arterial elastance and to predict arterial pressure response to fluid administration. Anesth Analg. 2015;120(1):76-84.
12. Monge Garcia MI, Guijo González P, Saludes Orduña P, Gracia Romero M, Gil Cano A, Messina A,et al. Dynamic Arterial Elastance During Experimental Endotoxic Septic Shock: A Potential Marker of Cardiovascular Efficiency. Front Physiol. 2020;11: 562824.
13. Monge García MI, Guijo González P, Gracia Romero M, Gil Cano A, Rhodes A, Grounds RM, et al. Effects of arterial load variations on dynamic arterial elastance: an experimental study. Br J Anaesth. 2017;118(6):938-946.
14. Lee JH, Kwon YL, Na JH, Jang YE, Kim EH, Kim HS, et al. Is dynamic arterial elastance a predictor of an increase in blood pressure after fluid administration in pediatric patients with hypotension? Reanalysis of prospective observational studies. Paediatr Anaesth. 2020;30(1):34-42.

#
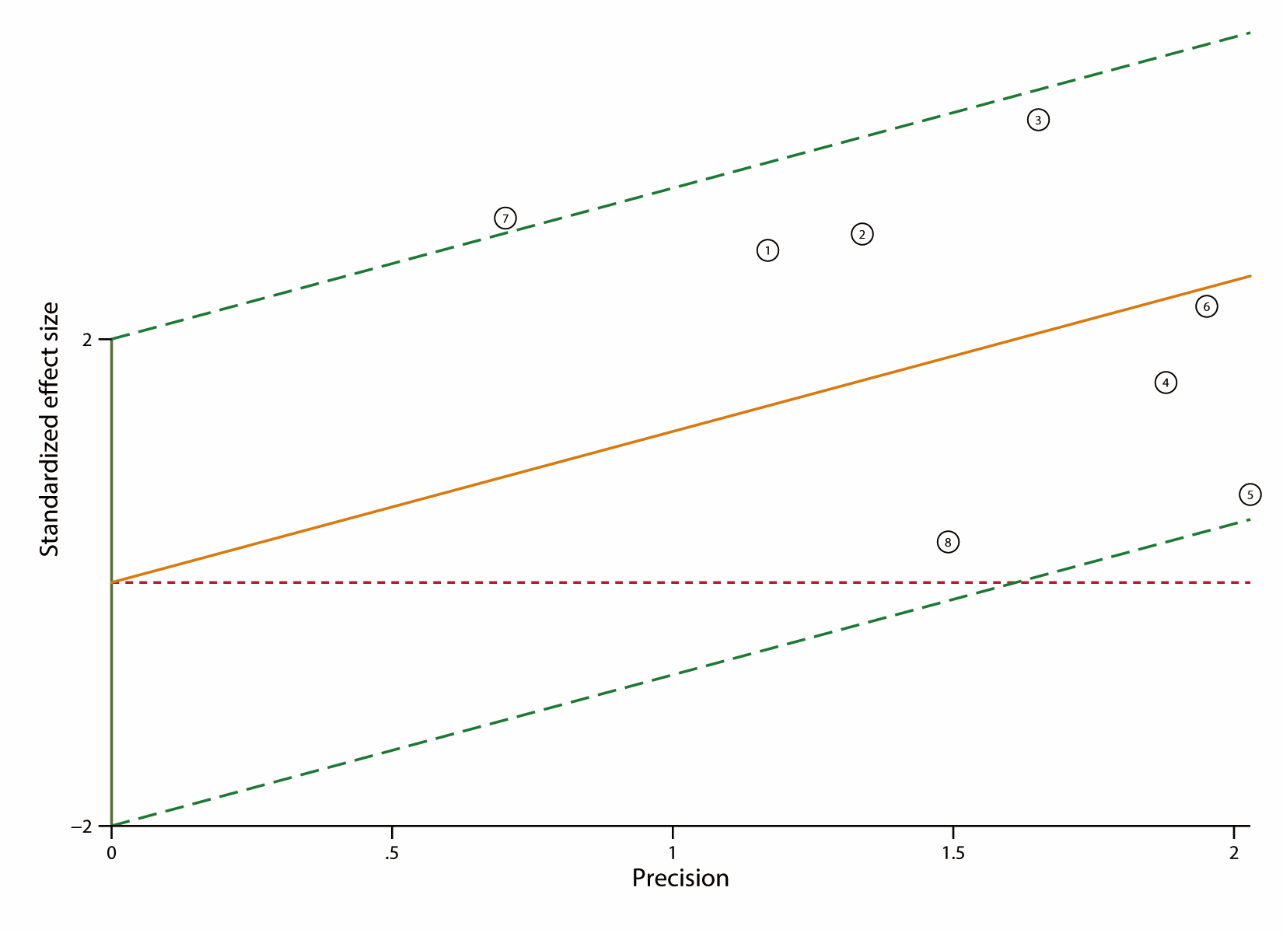
Figure S1. Galbraith plot to identify the outlier

Study No.7 represents the study by Guarracino et al.

# Figure S2. Bayes nomogram of dynamic arterial elastance for the prediction of mean arterial response to fluid expansion


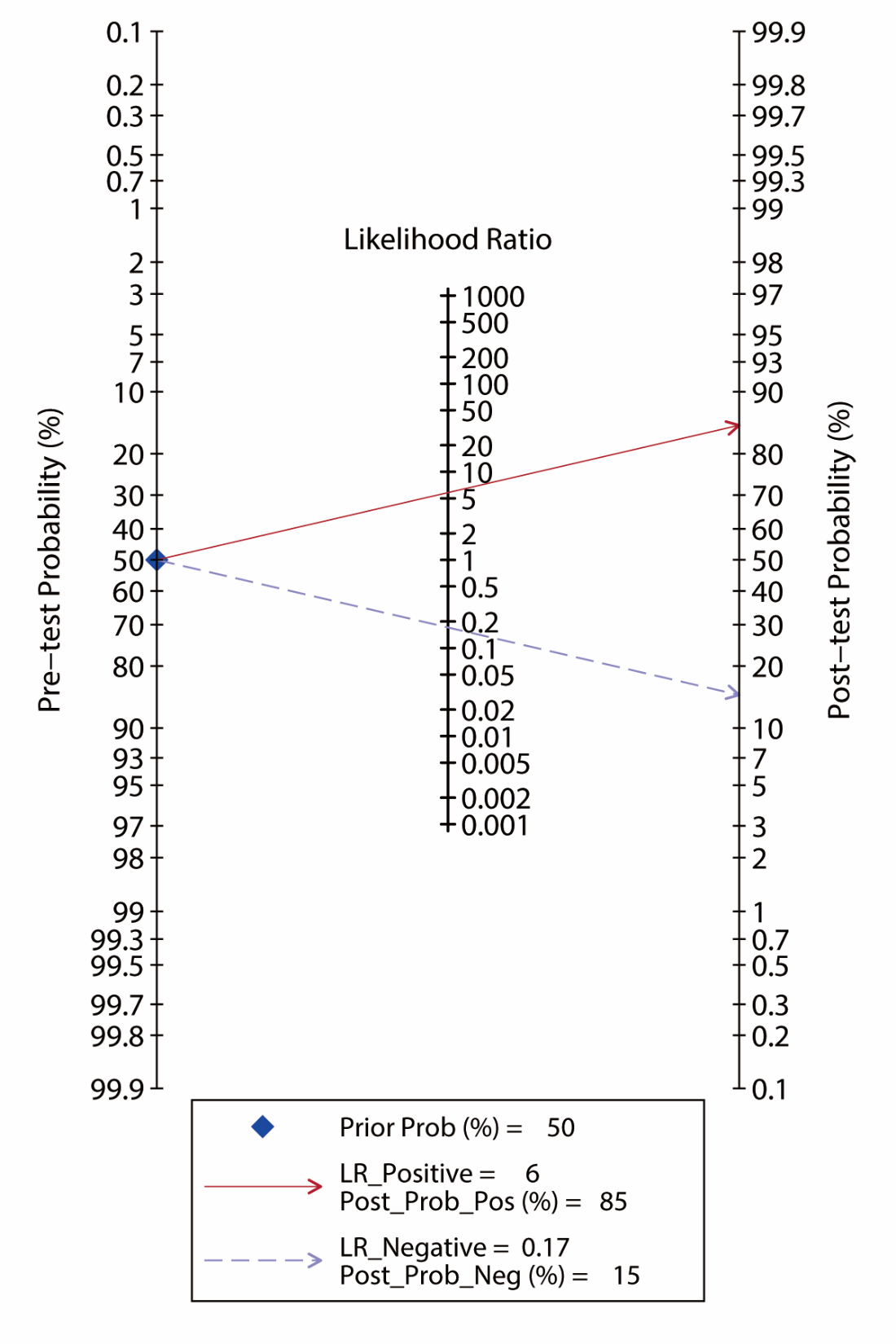


#
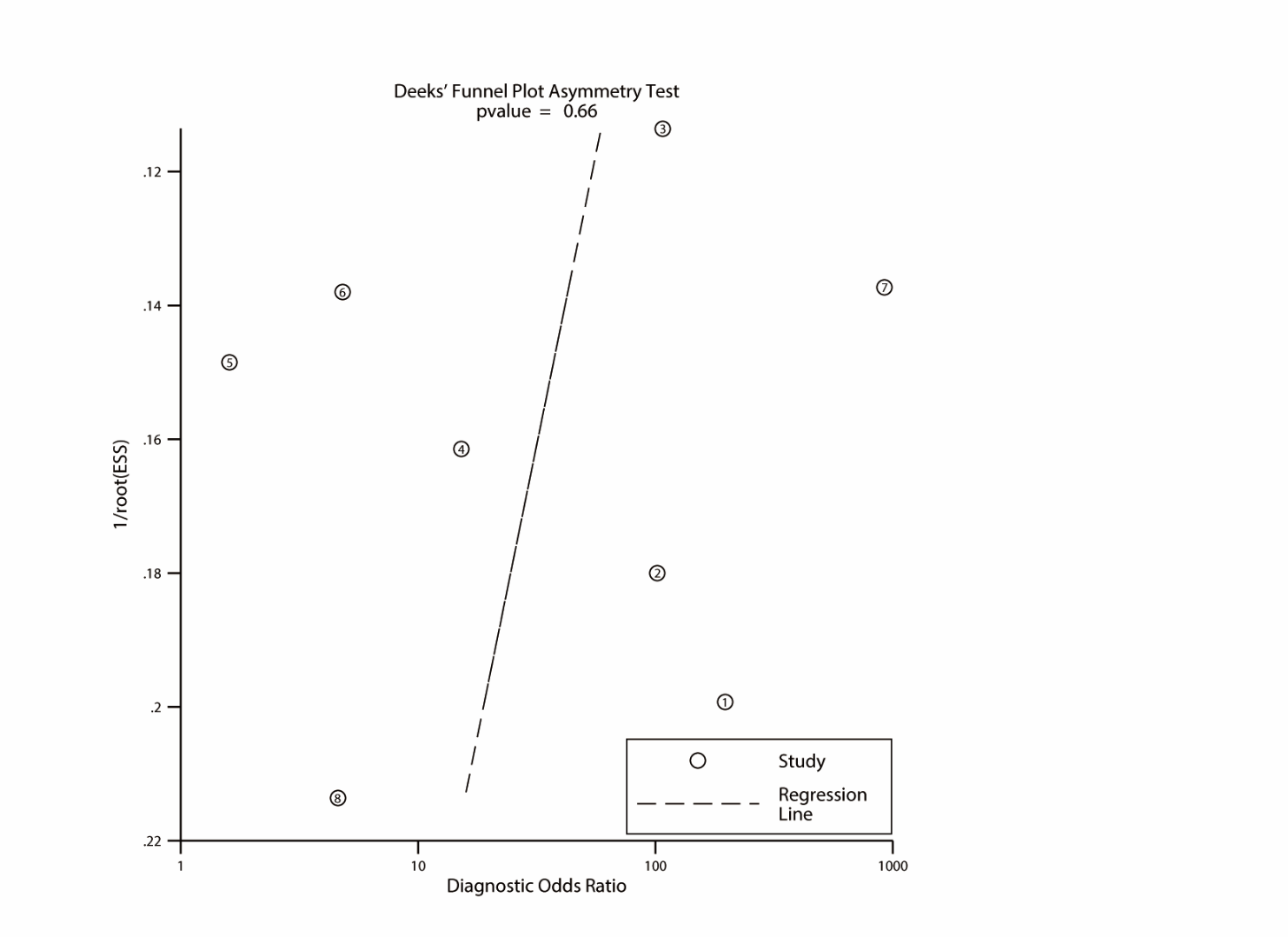
Figure S3. Deeks’ funnel plot to assess the publication bias
